# On Syphilitic Ulcers of the Larynx

**Published:** 1823-04

**Authors:** Cæsar H. Hawkins

**Affiliations:** Member of the Royal College of Surgeons.


THE LONDON

### Medical and Physical Journal.

4 OF VOL. XLIX.] APRIL, 1823. [No. 290.

For many fortunate discoveries in medicine, and for the detection of numerous errors, the world is indebted to the rapid circulation of Monthly Journals; and there never existed any work, to which the Faculty, in Europe and America, were under deeper obligations, than to the Medical and Physical Journal of London, now forming a long, but an invaluable, series.—RUSH.

### ORIGINAL COMMUNICATIONS,

### SELECT OBSERVATIONS, &c.

ART. I.—*On Syphilitic Ulcers of the Larynx.* By CÆSAR H. HAWKINS, Member of the Royal College of Surgeons.

THERE are few diseases so frightful to the bystander, and so formidable to the patient himself, as those to which the larynx is subject. The dread, and indeed the actual danger, of suffocation is so urgent, that it is not surprising that the disorders which produce so distressing a state should have long engaged the anxious attention of the physician. But, while so much has been done in the investigation of some other affections of this organ, it is somewhat remarkable that scarcely any attention has been paid to the subject of ulceration of the larynx.

This neglect is particularly striking in the numerous systematic works that have been devoted to the consideration of syphilis, as it is a frequent and very fatal symptom of this disorder. Perhaps it may be objected, that ulcers in the larynx are not in reality of venereal origin; that they will rarely, if ever, occur, where the venereal disease has been allowed to run its career unchecked. That ulcers in the larynx are sometimes the result of common inflammation in this part, cannot be doubted, as they have been found in children who have died after inflammation of the air-tubes; but, as the following observations were chiefly made during a residence as house-surgeon at the Lock Hospital, the affection of the throat has, in almost all the cases I have witnessed, been accompanied by some other venereal symptom, or the patients have suffered from the previous ravages of the venereal virus; and if so, they as well deserve the name of venereal as many other diseases which are usually attributed to its baneful influence. I have, however, seen cases of ulcerated larynx, where the history of the previous symptoms, the time which had elapsed since the reception of the primary symptoms, or some other circumstances, have afforded strong grounds of belief that the disease is strictly syphilitic, even confining the term exclusively to the symptoms which follow that peculiar primary sore, to which the name of chancre has been exclusively given.

The symptoms of ulcerated larynx in some measure resemble those of idiopathic inflammation: there is however this essential difference, that, instead of arising in persons of robust health and inflammatory diathesis, they appear, for the most part, to occur in persons originally of languid and unhealthy constitutions, or whose health has been undermined by the ravages of the venereal virus, or its scarcely less formidable antidote.

The disease of the larynx is most frequently the consequence of previous ulceration in the superior part of the fauces, and it is probable that any sloughing ulcer of the throat may extend downwards to the cartilages of the larynx; but there are principally three kinds of ulcers in the throat, which are apt in this manner to affect the larynx. One of these ulcers is very rapid in its progress, and of a more acute form; the second, and most common ulcer, often takes some weeks, or a still longer period of time, before it arrives at any formidable height: hence they may be denominated, respectively, the acute and the chronic sloughing ulcers of the throat. The third, and least common kind of ulcer, may be called, from the peculiar mode of its appearance, the painful sloughing ulcer.

The progress of these ulcers may naturally be divided into three stages: in the first of which the ulceration is confined to the fauces; in the second it has extended to the larynx; and, if it be not fatal in one or other of these stages, the lungs or trachea become implicated in the mischief, and death, in this third stage, terminates the sufferings of the patient.

The first description of ulcer in the throat, which is apt to spread to the larynx, begins in the tonsils, in which a deep ulcer will be seen, containing a thick black slough, which so rapidly extends, that it deserves the name of the acute sloughing ulcer. The black sloughs do not separate and successively form, as is sometimes the case in the other species, but portions of the sound parts become every successive day involved in the slough, which hangs by shreds from the palate and pharynx; the secretion of saliva being very much increased. The countenance is, almost from the beginning, bloated, and of a dark venous hue, before the symptoms of ulcerated larynx begin, and consequently before the blue colour can arise from any obstruction in the larynx itself. It rather appears to originate in the excessive languor of the circulation; a debility which it is impossible to remove, and which is such that the act of respiration itself appears to be too great a labour for the muscles to sustain. The slow passage of the blood through the lungs produces a copious secretion of mucus from the air-cells, which threatens to put an entire stop to the circulation. If the epiglottis and arytænoid cartilages become affected, the destruction of the patient is inevitable. The throat is so excessively irritable, that not the slightest stimulus can be borne. The cells of the mucous membrane become gorged with serum, and fill up the aperture of the glottis; the parts having very much the appearance of an erysipelatous affection, to which disease this kind of ulceration really bears considerable resemblance, both in its progress and termination. The death may sometimes occur without the acute symptoms of ulcerated glottis, so that the patient may sink gradually till within the last few minutes.

The second, or chronic ulcer, occurs chiefly in persons who have used mercury for this or some other form of the venereal disease; so that, perhaps, some doubts may be felt as to the origin of this species, even by those who grant the syphilitic nature of the acute kind. But, as few persons have suspicious symptoms of any severity without having taken mercury in some stage of their progress, except in situations where they are completely under the control of their medical attendants during the whole progress of the disease, the same doubts might be felt with regard to almost every other symptom of the venereal disease, particularly those usually called syphiloid or cachectic, if they had not each of them occasionally been observed after primary sores, for which no mercury had been exhibited. These observations particularly apply to the lower orders in the metropolis. It is not, however, of material importance to attach a name to the ulcer, as the employment of mercury is, in general, wholly inadmissible.

This ulcer consists of a slough of a yellowish-brown colour, beginning generally in the pharynx, just behind the posterior arch of the palate, or else immediately behind the tonsil, on one or both sides. The slough is smooth and uniform in its appearance; it is not very deep, and the edges are on the same level with the surrounding soft parts. If it is on the increase, the immediate edge of the sound part has an irritable appearance, with a mixture of red and yellow streaks or spots, the red lines being gradually lost, so as to enlarge the size of the slough, without any apparent breach of continuity between the slough and the healthy pharynx. The local symptoms attending this ulcer in the pharynx are very frequently of an extremely mild nature, so that the whole pharynx may be covered with dark yellow sloughs, while the patient complains of little more than an uncomfortable sensation in swallowing, not actually amounting to pain; for, in fact, the ulceration itself is not the cause of pain, so much as the contact of the food with the ulcerated surface, which cannot take place till the healing process has begun, and the separation of the sloughs has commenced. But, in general, although the patient himself does not suffer very much, his system sympathizes with the extensive destruction which has ensued: the countenance is that of general ill health, with an exceedingly quick, weak, and irritable pulse, loss of appetite, broken unquiet sleep, and very great emaciation and debility.

The last kind of ulcer which I shall describe is preceded, for two or three weeks before the appearance of any ulceration, by most excruciating pain, generally situated in the posterior part of the pharynx about the centre; the redness and other inflammatory appearances being very slight, but the difficulty in swallowing so very great that the patient's strength can scarcely be supported. At last a deep circular ulcer is perceived, which rapidly destroys the muscular part of the pharynx by sloughing, resembling the acute ulcer in appearance, and in being unaccompanied with any suppuration, but with more dryness of the mouth, and a fever, more of the inflammatory than of the typhoid character. In one case, the acute pain was followed by the appearance of several little ulcers, which very quickly spread into each other. I have not had an opportunity of observing this last kind of ulcer, except in its first stage: I have been informed, however, on authority which I cannot doubt, that it is liable to extend to the larynx, though not so frequently as the other two which I have described.

Allowing for some slight differences, arising from the degree of acuteness, the symptoms of the second stage are so nearly alike, from whichever of these ulcers the larynx is affected, that I shall describe, chiefly, those which arise from the second; as, from its chronic nature, the symptoms are more easily marked, and the disease necessarily more easily cured.

With such symptoms as those which have been enumerated, the ulceration gradually proceeds till the epiglottis and arytænoid cartilages become involved in the disease. It is, however, somewhat remarkable, that the severity of the symptoms does not gradually increase in proportion to the extent of the ulcer, but continues of nearly the same degree of violence till the larynx is ulcerated; and then a fresh series of symptoms suddenly appear, constituting what I have called the second stage of the disease. For instance, a woman had extensive ulcers in the throat, which resisted every mode of treatment for many months. Nothing could rouse her constitution from the excessive languor into which it had sunk: but suddenly some accidental cause, such as a crumb of bread having fallen into the glottis, which had probably been some time diseased, caused a fresh activity in the ulcer, and gave rise to a new set of symptoms, which were fatal in a few days.

At this period the voice loses its sonorousness, so that it becomes little more than a whisper, each attempt to converse producing a suffocating cough. Each act of inspiration is attended with a peculiar rattling noise, (different, however, from that of croup,) while that of expiration is comparatively easy. The muscles of the larynx and os hyoides are in constant and violent action, so that the larynx cannot be held steady for an instant; and at every inspiration the head is elevated, as if to give greater freedom to the entrance of the air, by straightening the tube through which it has to pass. If the difficulty of breathing be very great, the arms are tossed about in rapid and unceasing motion; while the eyes are fixed and projecting, or drawn up under the upper eyelid, so as to hide the pupil. The sleep is sparing, and broken by starts, with the dread of impending suffocation. The countenance is generally pale, and expressive of the greatest anxiety; the face being covered with a greasy secretion, or bathed in profuse perspiration. The pulse is rapid in the extreme, so as scarcely to be counted; it is weak and excessively irritable; while the whole nervous system appears to be sunk in the depth of languor and debility.

If the disease is accompanied with inflammation of the chest, the state of the pulse is liable to be affected. In one case, where there were repeated attacks of inflammation, the pulse, although weak in general, had the peculiar wiry feel of active inflammatory action; and in another, it was even full and hard. Such a combination of disease is particularly dangerous, as the treatment necessary for one may be improper for the other affection.

But the most marked symptoms are those which affect the larynx itself. These are excessive pain and tenderness of the larynx, not general, but most commonly so far circumscribed that it is possible to distinguish whether the thyroid or cricoid cartilage is affected, and even which side of each individual cartilage: indeed, it is sometimes possible to cover with the point of the finger the spot which is the seat of the ulceration, as I have ascertained by dissection. When solid food is swallowed, it produces some degree of pain, but inferior to that of an acute inflammation of the pharynx, and in general is swallowed without material uneasiness; but the attempt to swallow fluids is invariably followed by acute pain and severe cough, arising, no doubt, from the insufficient closure of the glottis, in consequence of the morbid state of the muscles which regulate its actions; the irritation not being material from the deglutition of a solid morsel. Mr. Shaw, however, has recorded a case of ulcerated larynx, in which there was so much difficulty of swallowing that it was treated as a stricture of the œsophagus, till the employment of an opiate proved the difficulty to have been merely spasmodic.* I have heard the difficulty of deglutition remarked as a distinction between ulceration of the larynx below the glottis, and an ulcer involving also the parts above the chordæ vocales,—that the difficulty exists in the latter case, but is not present in the former. That the difficulty of swallowing is greater where the pharynx is ulcerated, must be evident; and perhaps there may be none where the ulcer is below the glottis. The circumstance, however, to which I would attach importance, is not a difficulty of deglutition, but the fact of a few drops of fluid almost always entering and irritating the glottis; the rest of the liquid being swallowed without any effort. The fluid enters the glottis in cases where the epiglottis is entire, and persons have been known to swallow with tolerable ease after it has been destroyed; facts which might be expected from the experiments of Majendie, and which prove that it is the affection of the muscles which produce the irregular deglutition, and not the loss of the mechanical assistance of the epiglottis.

Such are the most prominent symptoms of an ulcer in the larynx; they are constant, but vary in their intensity at different times; and their severity is by no means proportionate to the extent of the ulceration in different individuals.

There is one affection of the larynx which occurs so long after the preceding ulceration of the upper part of the throat, that it cannot always be traced to it, and perhaps may sometimes occur as a specific disease of the larynx. It is well known that the larynx is not unfrequently found to undergo a change, similar to that which takes place in the arteries of all old persons, the whole of the cartilages being sometimes converted into bone. The disease, however, to which I allude is the change of part of one of the cartilages into bone, occurring in persons before the whole system has acquired that state which disposes to the deposition of ossific matter, and appearing, therefore, to arise from some local action. It is possibly the result of long-continued inflammatory action of a mild nature. I have only happened to see two cases of this nature, though I have been informed of several others. In one, the greater part of the cricoid cartilage was changed into bone, some of which lay loose and carious in an ulcer of considerable extent. In the other, a man who died in St. George's Hospital, there was an abscess in the left side of the thyroid cartilage, very near the sacculus laryngis, containing several pieces of exfoliated bone. The symptoms in these cases appear to be very nearly the same as in the common ulcers of the larynx, unless there is a difference in the voice, which has a hoarse raucous sound. In the

* *London Medical and Physical Journal*, vol. xlviii. page 184.

last of the cases above mentioned, there was, it is true, no actual ulceration of the inner membrane of the larynx; yet the proximity of the abscess to the glottis will easily account for there being the same excitement of the muscles.

Mr. C. Bell* has mentioned the suffocation arising from hysteria, as in some measure like that of an ulcer in the larynx: a very little observation, however, of the countenance will show the trifling similitude between the two diseases; but the symptoms which have been enumerated as belonging to ulcerated larynx bear so much resemblance to those of cynanche laryngea, that it is of importance to point out some essential differences, in order to establish an accurate diagnosis between the two diseases; particularly as it often happens that there may be no ulceration in the throat high enough to be apparent on inspection. But, although a view of the ulcer may be denied us, we can sometimes ascertain whether one be present, by examination as far as the rima glottidis. If the finger is pressed firmly on the tongue, so as to avoid titillation, it may be passed round the epiglottis, and borne a sufficient time to ascertain the existence of ulceration, by the roughness or other deviation from the natural surface of the part. There are also some differences when the ulcer is below the rima glottidis. In cynanche, there is not the circumscribed tenderness of ulceration; there is much more pain in the deglutition of solid substances; and the swallowing fluids does not excite the peculiar action of the muscles of the glottis, or not in the same degree; there is also a difference in the voice.

But one of the chief diagnostic marks between ulceration and simple inflammation of the larynx, is perceptible in the state of the circulation. The pulse in the latter, as in almost all acute inflammations, is full and hard; the countenance flushed, and the eyes suffused with blood; "febris acutà et fere ardens, facie florida, deinde livescens." The change of colour does not begin till quite the last stage of a very acute disorder rapidly coming to a crisis; the reverse of all these circumstances generally obtaining in ulceration of the glottis. It is true that, in the latter stages of inflammation, it may be impossible to distinguish between them, or any other affection of the respiratory organs, a short time previous to death; but, in nearly all the numerous cases of cynanche laryngea recorded in the Medico-Chirurgical Transactions, where the medical attendant was called in early, large bleeding was the first remedy which occurred to his mind, from the evidently increased force of the circulation.†

---

* *Surgical Observations*, part i. page 40.

† See particularly vol. vi. pages 138 and 148.

The larynx is also found in a different state after death: the tube being always more or less obstructed after simple inflammation, either by effusion beneath the mucous coat, and consequent thickening of the membrane, or, more rarely, by the deposition of a false membrane, as is always the case in cynanche trachealis. In ulceration, the difficulty of respiration arises much more from irritation than from any actual impediment to the entrance of the air; and in some specimens, so far from the glottis being obstructed, the whole larynx and trachea have been found of preternatural size, so that the finger might be passed through the glottis. The expectoration is also more purulent than in cynanche laryngea.

The ulcers in the larynx vary in size, from that of a split bean, or almond, to the diameter of an inch or more. The side of the arytænoid cartilages appears to be their most frequent situation, as might be expected from their origin behind the tonsils. The epiglottis is, however, sometimes alone affected; and the largest ulcer I have seen was situated in the posterior part of the cricoid cartilage and sides of the thyroid. In one case, the ulceration had eaten away the upper half of both arytænoid cartilages, and laid bare the cornua of the os hyoides and thyroid cartilages: the horns of the former, having been deprived of vitality by this means, were of a black colour, and projected into the œsophagus. The epiglottis is sometimes wasted, or even completely destroyed. An abscess round the larynx sometimes involves the larynx itself in destruction: in such cases, the arytænoid cartilages not unfrequently slough off, or are found about to do so after death.

The ulcer in general has a ragged, irregular surface, the deeper parts being thickened so as in some measure to supply the loss of substance occasioned by the ulceration. If the tonsils have not been lost previous to death, their ducts are seen much enlarged, and so irregular that to a superficial observer they will have the appearance of ulcers.

The third stage of laryngeal ulceration is when the lungs and trachea become affected.

The difficulty of respiration produces so great a degree of fulness of the vessels of the lungs, that a quantity of watery fluid is poured out, which completely prevents the purification of the blood. Sudden death from this cause is not uncommon: hence it has been called, by some pathologists, an apoplexy or dropsy of the lungs. It has already been mentioned, that a degree of active inflammation is occasionally excited, which materially interferes with the treatment necessary for the ulcers.

By far the most frequent sequel of ulcers in the larynx, is, however, the common tubercular phthisis of the lungs, the intermediate parts of the trachea and bronchia being free from ulceration. Whether phthisis pulmonalis is actually produced by the laryngeal ulcer, or whether it only excites a latent predisposition to tubercular formation, it is not of material importance to determine. A modern writer* has, indeed, actually given the name of venereal to the consumption which occurs in syphilitic patients, and has proposed the employment of mercury for its cure. It is sufficient, however, for our present purpose to observe, that symptoms of consumption will often show themselves after an attack of ulceration in the larynx, and most rapidly terminate by the dissolution of the patient.

[From the length of the Original Communications, we regret that we are under the necessity of deferring the rest of Mr. HAWKINS's paper till next month.]

* M. LAGNEAU. See *Foreign Quarterly Journal*, vol. iv. p. 529.

